# T-Cell Lymphoblastic Lymphoma Showing Aberrant Synaptophysin Expression in a Child

**DOI:** 10.4274/tjh.galenos.2019.2019.0307

**Published:** 2020-02-20

**Authors:** Nazım Emrah Koçer, Bermal Hasbay, Fazilet Kayaselçuk

**Affiliations:** 1 Başkent University Faculty of Medicine, Adana Dr. Turgut Noyan Research and Application Center Department of Pathology, Adana, Turkey

**Keywords:** Pediatric leukemias, Pediatric lymphomas, Acute lymphoblastic leukemias

## To the Editor,

The term “malignant small round cell tumor” represents a highly aggressive group of tumors comprising small, monotonous, relatively undifferentiated cells with high nucleus/cytoplasmic ratios [1]. Despite histopathologic similarities, the treatment modalities of each tumor in this generic group are different than the others and differential diagnosis is crucial. Histopathologic examination generally needs to be supported by ancillary techniques, most commonly and practically by immunohistochemistry, for the correct differential diagnosis. Immunohistochemically aberrant expression of an antigen in a small round cell tumor may cause a diagnostic dilemma.

Herein we report a case of T lymphoblastic lymphoma in a 9-year-old child showing aberrant synaptophysin expression.

**Figure 1:**
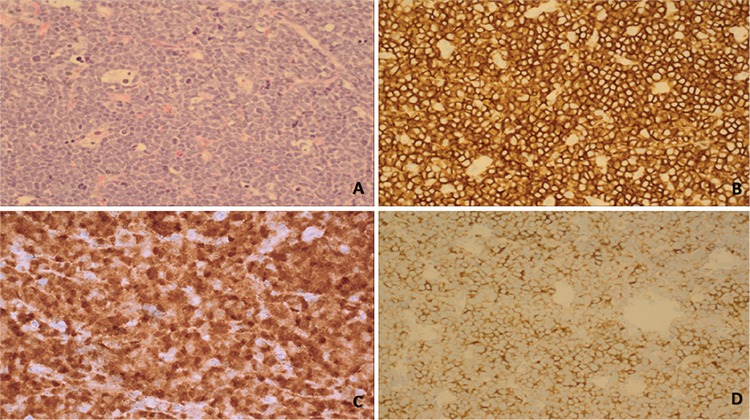
Microphotographs reveal: A) diffuse infiltration of small cells with hyperchromatic nuclei, scant cytoplasm, and monomorphic atypia (H&E, 400^x^); B) strong membranous immunohistochemical CD2 staining (CD2, 400 400^x^), C) diffuse nuclear TdT reactivity (TdT, 400 400^x^); and D) immunohistochemical synaptophysin expression (synaptophysin, 400 400^x^).

A 9-year-old female patient was admitted to the hospital with cough, dyspnea, and a mass in the left thoracic region. Computerized tomography revealed a mass in the anterior mediastinum; right paratracheal, hilar, and subcarinal lymphadenomegalies; and severe left pleural effusion. Scanning results of other regions were within normal limits. A Tru-Cut biopsy was taken from the mediastinal mass. Histopathological examination revealed a diffuse infiltration composed of small cells with hyperchromatic nuclei and scant cytoplasm, showing monomorphic atypia. In immunohistochemical study the tumor cells were positive for LCA, CD2, CD3, CD10, CD1a, TdT, and synaptophysin (Figure 1), while CD20, pancytokeratin, CK-19, chromogranin A, and CD56 were negative. The Ki-67 proliferation index was higher than 90%. There was no bone marrow infiltration.

The patient was diagnosed with T-cell lymphoblastic lymphoma. Following adequate treatment, the patient is doing well in her third year after the diagnosis.

Small round cell tumors most commonly affect the pediatric age group and represent a variety of tumors, including non-Hodgkin lymphomas, Ewing/primitive neuroectodermal tumor (PNET), neuroblastoma, small cell osteosarcoma, rhabdomyosarcoma, synovial sarcoma, hepatoblastoma, nephroblastoma, and retinoblastoma [1]. Although each tumor needs different management strategies and therapeutic modalities, due to the histopathologic similarities and frequently overlapping clinical presentations, differential diagnosis can be challenging.

Histopathologic differential diagnosis requires a careful assessment of slides for specific morphological features of each tumor and the evaluation of an adequate immunohistochemical spectrum by using thoroughly optimized antibodies. Aberrant expression of an antigen may lead to misdiagnosis in cases in which well-established diagnostic flow charts for immunohistochemical profiles are not followed.

Expression of a neuroendocrine marker in lymphoma is extremely rare. To our knowledge, there are only two cases of synaptophysin expression reported in lymphoblastic lymphoma. The first case was reported by Alvaro et al. [2], in a 6-year-old girl, and the second case was reported by Patel et al. [3], in a 42-year-old male patient. Both of these patients also had jaundice in common as a presentation symptom. In our patient the disease was limited to the mediastinum and there was no sign of jaundice. The favorable prognosis suggests that aberrant expression of synaptophysin had no prognostic impact in our patient.

The most common synaptophysin-positive small round cell tumors are PNET and neuroblastoma in childhood [1]. A proper immunohistochemical panel covering major entities in differential diagnosis and enabling cross-checks between antigen expressions, combined with careful histopathologic assessment, can prevent a misdiagnosis.
